# Edible bamboo shoots: a sustainable nexus of nutrition, livelihood, and food security for rural communities in India

**DOI:** 10.3389/fnut.2025.1654510

**Published:** 2025-12-03

**Authors:** Pradip Kumar Sarkar, Bijoya Bhattacharjee, Sujit Das, Bapi Das, N. Raju Singh, Pempa L. Bhutia, A. Chakrabarti, Bikash Das, Rubai Podder, Burhan U. Choudhury

**Affiliations:** 1 ICAR Research Complex for NEH Region, Tripura Centre, Lembucherra, West Tripura, India; 2 ICAR Research Complex for NEH Region, Shillong, Meghalaya, India; 3 ICAR Research Complex for NEH Region, Nagaland Centre, Nagaland, India; 4 ICAR Research Centre on Litchi, Muzaffarpur, Bihar, India

**Keywords:** bamboo shoot, sustainable agriculture, nutritional, medicinal, cultural values, ecosystem services, bamboo based agroforestry, north east India

## Abstract

Bamboo, a fast-growing, durable, and versatile natural resource, plays a significant role in the livelihoods of rural communities in India by providing economic, ecological, and nutritional benefits. With its multifaceted utility, bamboo contributes to poverty alleviation, biodiversity conservation, and ecosystem restoration. While bamboo culms find extensive industrial use, edible bamboo shoots, particularly in the northeastern and eastern regions of India, have emerged as a vital food source rich in macro- and micronutrients. Species such as *Bambusa balcooa, B. bambos, B. vulgaris, Dendrocalamus asper, D. hamiltonii, D. giganteus*, and *Melocanna baccifera* are highly valued both for their edible shoots and ecological services. Despite challenges like cyanogenic toxicity, short shelf-life, and market linkages, improved processing techniques, fermentation practices, and agroforestry-based cultivation offer immense potential for sustainable agriculture, livelihood enhancement, and climate-resilient food systems. This review highlights the significance of edible bamboo shoots as a sustainable bridge between nutrition, livelihood, and food security in India, along with technological interventions and policy support needed to maximize their potential.

## 1 Introduction

Bamboos, belonging to the family Poaceae, are recognized as one of the fastest-growing, durable, and versatile natural resources, widely distributed across tropical and subtropical regions (1). Their utility spans societies globally, with rural communities in India deriving considerable economic and ecological benefits (2). Native to all continents except Europe and Antarctica (3), bamboos cover approximately 14.16% of India's forest area (4). India harbors 136 recorded exotic and indigenous species, with nearly 50% of total Bambusa species (5) and 66% of the national bamboo growing stock found in the Northeastern states (6, 7).

Bamboos are renowned for their multipurpose utility, surpassing many other multipurpose tree species (MPTs), and are often termed “cradle to coffin timber.” However, deforestation, anthropogenic pressures, and over-exploitation threaten bamboo resources, leading to ecological degradation (8–19). Bamboo-based agroforestry systems have emerged as practical tools for restoring degraded landscapes while providing economic returns to farmers (16). The medicinal applications of bamboo were referenced in Indian texts dating back around 10,000 years, highlighting its role in *Chyawanprash*, a health tonic rich in herbs including bamboo manna, known for its anti-aging benefits (20). Amid the diverse uses of bamboo, edible bamboo shoots are particularly significant for rural communities, serving as a source of nutrition, livelihood, and food security. However, to harness their full potential, scientific advancements in cultivation, processing, and value chain development are imperative.

In the nutrition composition and food fortification system, bamboo shoots have been fulfilling the criteria very effectively. The bamboo shoots are composed of lots of essential healthy fibers, vitamins, minerals, and other health-beneficial compounds. Generally, bamboo shoots are fresh plants (20–30 cm in length, narrow and pointed in shape, weighing more than 1 kg) with a soft, crisp, ivory-colored texture, consisting of a sheath, a stem (or tip), and a basal shoot. Usually, there are two types of bamboo shoots (winter shoots and spring shoots) available in a year, among which the bamboo shoots grown in spring are larger and more challenging than those grown in winter. Still, the latter shoots have good taste and rich nutritional values. However, the size and weight of bamboo shoots depend on several factors, including the location, depth, and nutrition of the soil, as well as irrigation and drainage conditions, climate, rainfall, temperature, and soil type and fertility. Fortified bamboo shoot products can be enriched with vitamin B and D, iron, and calcium to address micronutrient deficiencies.

Bamboo–based fortified food can be formulated for specific life stages, such as infant nutrition or elderly care. These products are being designed to support digestive health, boost immunity, or provide essential nutrients for growth and development (69, 70). Fortified bamboo shoot products can be marketed as functional foods, appealing to health-conscious consumers. The versatility of bamboo shoots allows them to be incorporated into various product formats such as powders, flakes, or canned goods. Bamboo-fortified foods can be tailored to meet the nutritional needs of different populations, including pregnant women, athletes, or individuals with specific dietary restrictions. By harnessing the nutritional benefits of bamboo, manufacturers can develop innovative products that promote overall health and wellbeing. These products can be fortified with probiotics to promote digestive health or Omega-3 fatty acids to support cardiac health simultaneously. This review aims to consolidate knowledge on the nutritional, medicinal, cultural, traditional, ecosystem service, and other essential uses of edible bamboo species in Northeast India. By integrating edible bamboo-based agroforestry system and their significance, there is need to (i) identify nutritional composition and its safety, (ii) assess the effects of harvesting ages and fermentation on nutritive qualities, (iii) to observe the socio-economic significance bamboo-shoots and market linkages of bamboo shoots in Tripura's economy, and also to highlight the knowledge key research and policy gaps of bamboo-based markets.

## 2 Methodology

### 2.1 Database formation and statistical analyses

**Figure 1:**
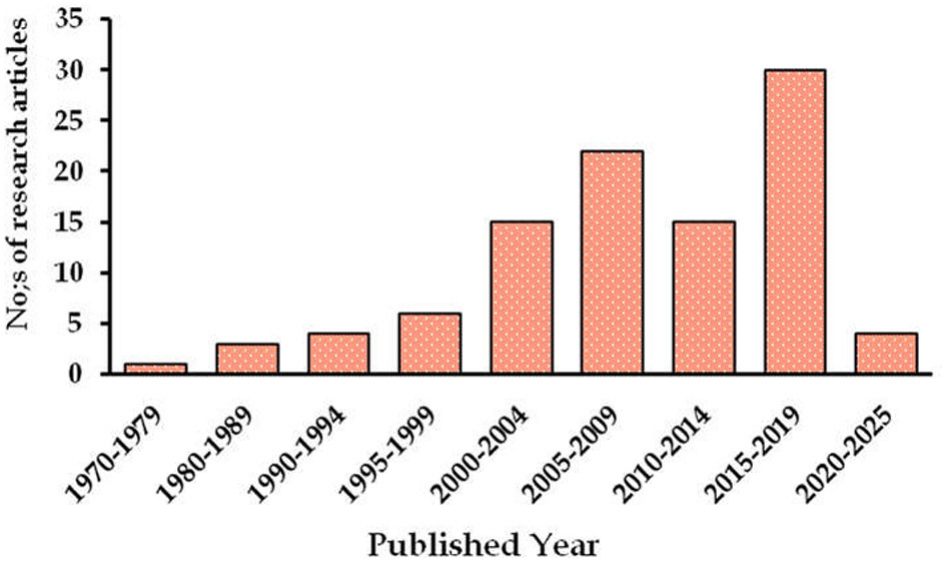
Year wise count of published research articles related to the bamboo and bamboo based research for the different parts of India.

A relevant set of published articles, government reports, scientific studies, and documents that address bamboo-based agroforestry, nutritional, and cultural. Traditional food security, socio-economy, and market linkages of bamboo or bamboo-based products for the NEI and other parts of India were identified and selected for the data synthesis through a literature search. To establish a database, we searched the ISI Web of Science (http://webofknowledge.com) and Google Scholar (https://scholar.google.com/) for peer-reviewed journals. Further, different combinations of keywords related to bamboo species practices/or bamboo products/or nutritive and medicinal compositions, ecosystem services, socio-economic significance, and challenges of edible bamboo shoots, etc. were used to search the related literature. First, a total of 180 records were identified by reading the titles of each publication and excluded 80 publications that were not related to the study area nor the objectives of the work. Second, the remaining 120 publications potentially relevant to the study were further scrutinized by reading the abstract of each record, and 100 papers were selected for writing this review article and its related data analysis. The bamboo shoots uses, bamboo-based AFS and socio-economy, as well as market linkages of bamboo-based products related data published during the last 47 years, between the period of 1978 to 2025, were included in the review, of which, more than 70% of the studies were conducted over the last 20 years, i.e., 2005 to 2025 (Figure 1). All the collected articles were screened to identify the most relevant research to meet the following criteria: bamboo, importance of edible bamboo and its scope, bamboo-based AFS, nutritional, cultural, medicinal values, ecosystem services, firewood's and their related parameters, significance of bamboo, nutritional composition of bamboo shoots and their safety, Effect of harvesting age and fermentation on nutritive qualities, socio-economy and its market linkages, etc., were followed during synthesis of this review article. Articles (original paper, review, synthesis) with their mean values, ranges, standard deviation, and standard error. Tables and graphs were used to represent the synthesized results.

## 3 Results and discussions

### 3.1 Importance of edible bamboos and scope

**Figure 2:**
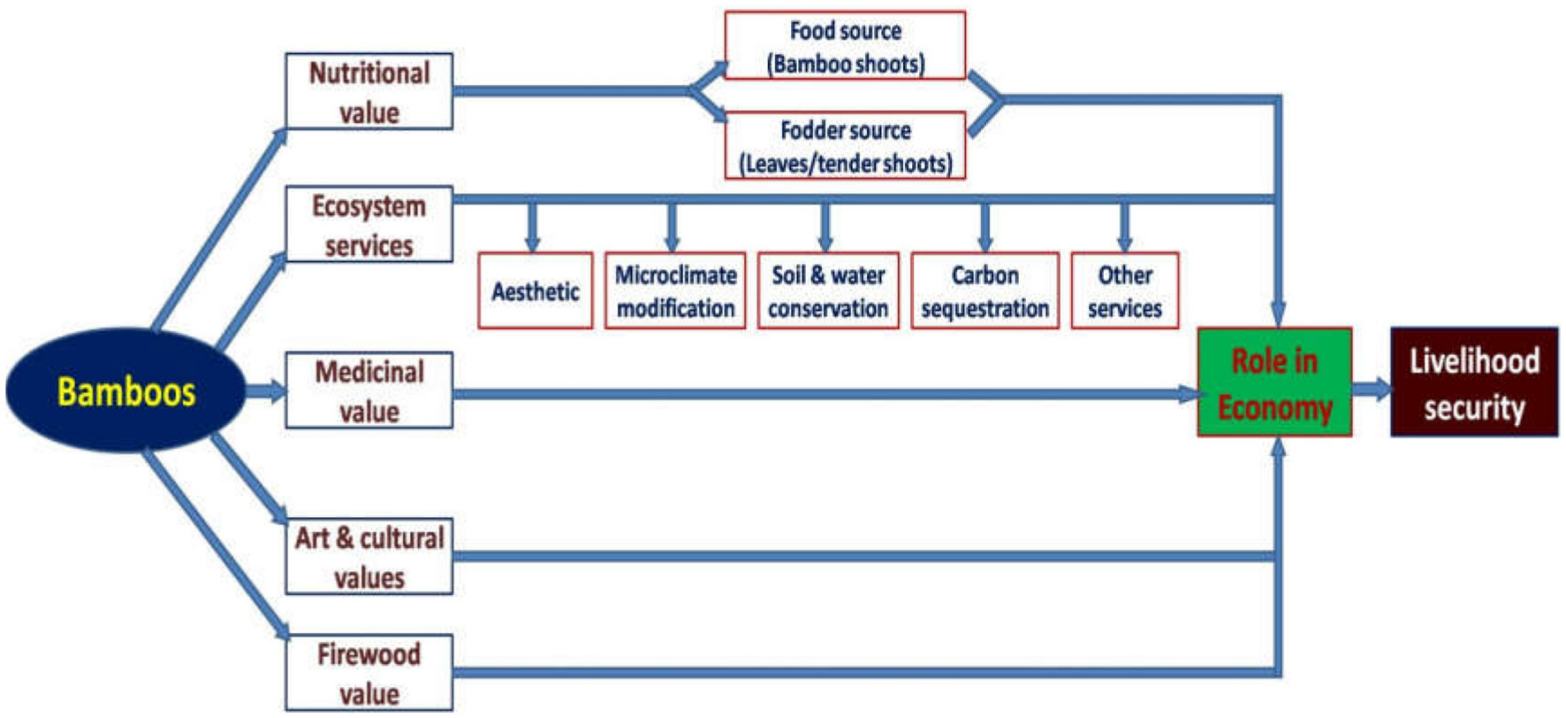
Importance and scope of edible bamboos in India.

The importance and uses of edible bamboos are summarized here under (Figure 2).

#### 3.1.1 Nutritional value

Bamboo shoots are a significant food source for rural communities. In several countries, including Japan, the United States, Thailand, Nepal, Australia, Bhutan, New Zealand, Korea, Malaysia, and Indonesia, as well as in India, bamboo shoots are considered a traditional delicacy among rural populations. Many ethnic groups consume bamboo shoots either as a vegetable or in the form of value-added products such as pickles or chutneys. For instance, in Indonesia, bamboo shoots are prepared with thick coconut milk and spices to make a traditional dish known as “Gulei Rebung.” In India, particularly in the state of Sikkim, people prefer to consume bamboo shoots in the form of a non-fermented curry, locally known as “tama” (21, 22). Similarly, people in the state of Jharkhand consume bamboo shoots either as a vegetable or in the form of pickles, prepared from raw, fresh, or fermented shoots. Various value-added products of bamboo shoots, including dry powder, small chips, fresh fermented pieces, and pickles, are widely available in the market. Many rural communities depend on bamboo shoots and their processed products for their livelihoods. Bamboo shoots are highly nutritious, containing a wide range of macro- and micronutrients, high fiber content, low calories, and low fat (7, 23). Due to these nutritional attributes, bamboo shoots have proven effective in reducing the gap between the demand and supply of essential nutrients in the human daily diet, thereby directly or indirectly contributing to achieving the national goals of food security, safety, and improved nutrition. In addition to their food value, bamboo leaves are regarded as excellent fodder for small ruminants such as goats (24) and sheep. A comparative study conducted by Jha and Chaturvedi (25) on the fodder quality of two species, namely *Bauhinia variegata* and *Dendrocalamus strictus*, revealed that *D. strictus* is superior to *B. variegata* in terms of nutrient content and crude protein levels. Consequently, farmers can cultivate bamboo either as block plantations, boundary plantations, or as part of bamboo-based agroforestry systems, which involve integrating bamboo with other agricultural crops and/or animals, to derive multiple benefits (16). Supporting this, a study conducted by Chandramoulia et al. (26) on five-year-old plantations of *Dendrocalamus asper* demonstrated that this species can be a profitable option for farmers if it is adopted appropriately.

#### 3.1.2 Ecosystem services

1. **Aesthetic:** Bamboo plantations play a crucial role in enhancing the greenery of degraded lands, wastelands, and non-arable areas. In addition to their ecological benefits, they have been reported to generate approximately 35% more oxygen compared to equivalent stands of trees (27).
2. **Microclimate modification:** Bamboo plantations significantly contribute to improving the microclimate by reducing light intensity, modifying soil and air temperatures, and altering relative humidity (28). Additionally, they offer protection against harmful ultraviolet (UV) rays and function as natural purifiers of both the air and atmosphere.
3. **Soil and water conservation:** Bamboos are highly effective in preventing soil erosion, owing to their extensive, net-like root systems and rhizomes that firmly bind the soil together. The combined stem flow rate and canopy interception rate for bamboo plantations is estimated to be around 25%, resulting in a considerable reduction in surface runoff and soil erosion (27). Research has shown that planting bamboo species such as *Bambusa arundinacea* can effectively control the rise in water tables, with recorded increases of 1.09 m, 1.86 m, 2.46 m, and 2.96 m during the first, second, third, and fourth years of growth, respectively (29). Furthermore, sediment yield has been reported to reduce to as low as 1.4 tons per hectare in high rainfall years, which is 10 to 20 times lower than that of untreated watersheds (30).
4. **Carbon sequestration:** Specific species, such as *B. cacharensis, B. vulgaris*, and *B. balcooa*, have demonstrated the ability to sequester between 1.20 and 1.46 tons of carbon dioxide per hectare per year (31). Additionally, other studies have revealed that cumulative soil carbon sequestration is maximized under bamboo plantations, with recorded values reaching up to 41 tons per hectare, which is substantially higher than those observed in fallow ravine lands (32).
5. **Other services:** In comparison to cotton fibers, bamboo fibers have been proven to provide almost 60% better protection against UV rays (27).

#### 3.1.3 Medicinal value

Bamboo shoots have been traditionally used as natural medicines in rural folklore across many countries, including India. They possess significant medicinal properties, including the ability to lower cholesterol levels, as well as antioxidant and anti-inflammatory activities (7). Due to these properties, bamboo shoots have been an integral part of Ayurvedic practices for centuries, notably serving as a key ingredient in formulations like Chyawanprash.

#### 3.1.4 Art and cultural values

Bamboos, being fast-growing and high-yielding renewable resources, serve as an essential source of raw materials for agroforestry products. A significant quantity of bamboo is utilized by plywood industries, where it undergoes thorough industrial processing to produce ply bamboo. This processed bamboo has gained substantial demand in recent times, especially for applications such as wall paneling, floor tiles, furniture making, briquettes for fuel, raw materials for housing construction, and rebar for reinforced concrete beams (33).

#### 3.1.5 Traditional uses of bamboos

The exceptional mechanical properties of bamboo, such as being 17% stronger in tensile strength than steel, 27% stronger than Red Oak, and 13% harder than hard Maple, combined with its lightweight and flexibility, make it a practical and viable alternative to many tropical timber species that are becoming increasingly scarce for furniture and building material industries (34, 35).

#### 3.1.6 Other uses

***a. Uses in forest-based industries*:** Bamboo serves as one of the finest raw materials for forest-based industries, with high demand for its use in the production of items such as incense sticks (agarbatti) and matchbox sticks.
***b. Use of bamboos in making musical instruments*:** In India, bamboo is traditionally utilized for crafting musical instruments, including the flute, locally known as Bansuri, and the Ektara, among others.
***c. Use of bamboo charcoal*:** Bamboo charcoal holds significant industrial value across various sectors. It is also preferred by goldsmiths for use in jewelry making due to its quality and specific properties.
***d. Defense value*:** In rural regions, bamboo plays a vital role in the preparation of defense tools such as bows and arrows, spears, lathi (sticks), and other protective implements. Villagers commonly use lathis as a means of defense against threats from wild animals, such as snakes and stray dogs.
***e. Uses of bamboo in agriculture*:** Bamboo finds extensive use in agriculture as handles for various implements, such as spades and axes. Additionally, many components of bullock carts are traditionally constructed using bamboo, highlighting its importance in rural transportation and farming operations.

### 3.2 Edible bamboo-based agroforestry systems and their significance

**Figure 3:**
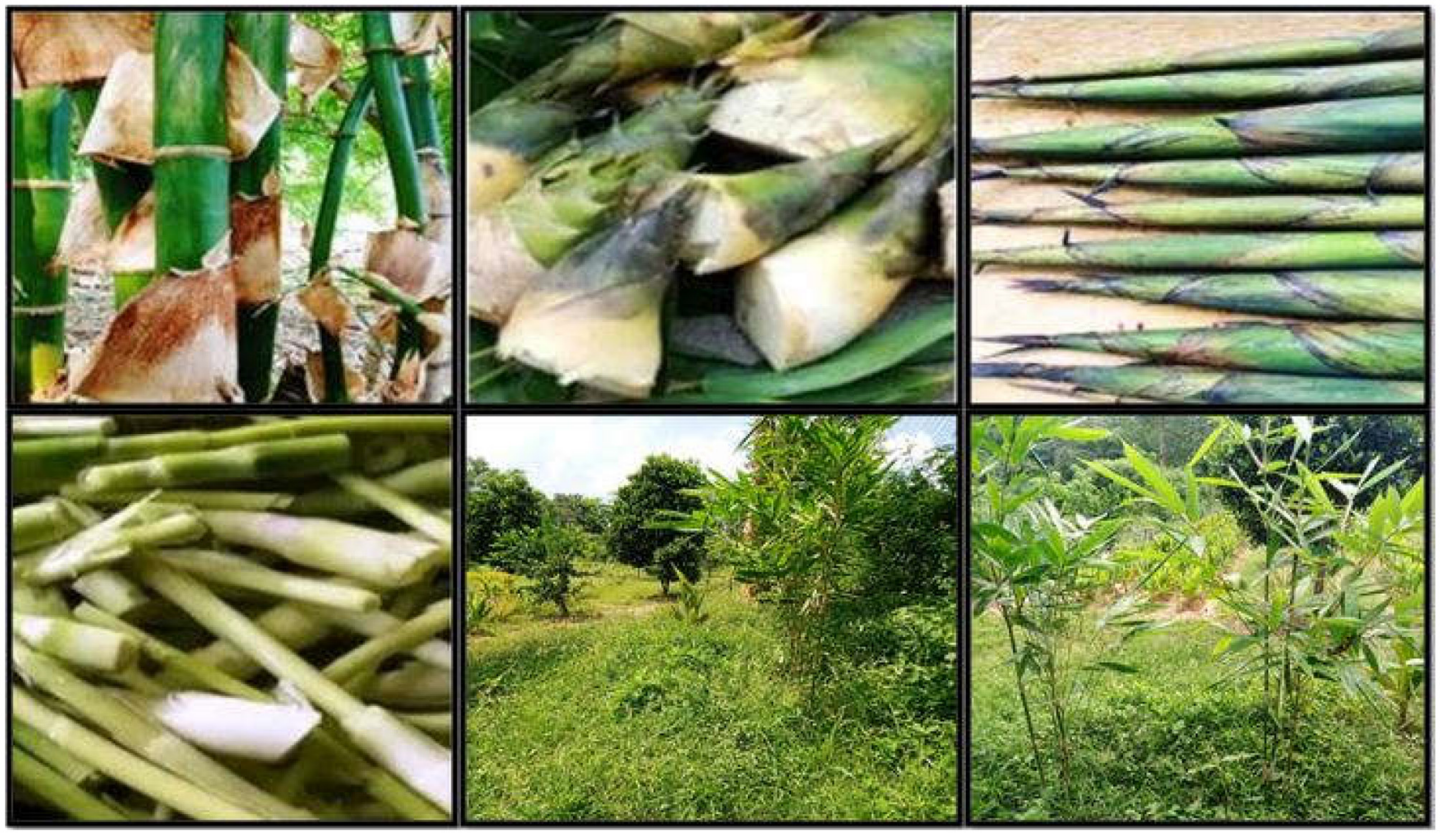
Physical appearance of bamboo-based agroforestry systems, bamboo clumps and bamboo shoots before and after processed as food.

Bamboo-based agroforestry systems are highly prominent in rural landscapes (Figure 3), where their adoption significantly improves ecological conditions, particularly on abandoned or degraded lands (36). Several studies have recommended increasing the spacing between bamboo plants to optimize space for alley cropping (6, 37). Banik (38) highlighted that cultivating shade-tolerant crops, such as ginger, turmeric, pineapple, and cinnamon, under mature bamboo clumps is both technically feasible and economically viable. In several regions, particularly in parts of India, bamboo species such as *Bambusa bambos, Bambusa nutans*, and *Dendrocalamus strictus* are grown in combination with alley crops like maize and soybean, enhancing both biodiversity and farm income. In the ravine areas of major river basins such as the Mahi, Chambal, and Yamuna, bamboo plantations have been successfully established using cost-effective management practices. For instance, harvesting one-third of the old culms per clump has been adopted as a sustainable approach to conserve natural resources and rehabilitate these degraded ecosystems (32, 39). Bamboo is also planted along the boundaries of agricultural fields, acting as effective shelterbelts or windbreaks to protect alley crops from frost and other climatic adversities (24). Notably, block plantations of *Dendrocalamus strictus*, aged 5 years and spaced at 5 m × 5 m, have been reported to yield a minimum Benefit-Cost (B:C) ratio of 2.07 at a 10% discount rate, with even higher returns achievable in subsequent years (34). Furthermore, Chandramoulia et al. (26) observed that a 5-year-old plantation of *Dendrocalamus asper* can achieve a minimum B:C ratio of 6.21 (at a 10% discount rate) based solely on shoot yield, making it a highly profitable option for farmers cultivating bamboo for shoot production.

A field experiment conducted at the research farm of the ICAR Research Complex for Eastern Region, Farming System Research Center for the Hill and Plateau Region, Ranchi, Jharkhand, yielded promising results for edible bamboo plantations (Table 1). Among eight edible bamboo species assessed in 2-year-old block plantations, *Bambusa tulda* var. Taral and *Gigantochloa atraviolacea* exhibited the highest total number of culms produced per clump. However, when average volume per culm was considered, *Bambusa balcooa* emerged as the superior species. These findings suggest that the selection of bamboo species for plantations should be tailored to the specific needs and objectives of the grower.

**Table 1:** Yield of culms per clump of 2 years old bamboo species grown under eastern plateau and hill region.

**Species**	**Number of culms per clump**	**Average volume per culm (Actual) in cm^3^**
*Bambusa balcooa*	4.33 ± 0.90^c^	4,726.27 ± 708.94^a^
*B. vulgaris*	7.20 ± 1.50^c^	1,216.4 ± 182.46^c^
*B. tulda* var. Taral	23.00 ± 3.68^a^	1,764.43 ± 264.66^c^
*B. balcooa* (Plus tree)	3.00 ± 0.48^c^	3,280.74 ± 492.11^b^
*B. arundinaceae*	9.60 ± 1.54^b^	104.15 ± 15.62^e^
*Dendrocalamus membranaceous*	7.80 ± 1.25^c^	724.64 ± 108.70^d^
*D. strictus*	6.00 ± 0.96^c^	530.21 ± 79.53^d^
*Gigantochloa atroviolacea*	17.90 ± 2.86^a^	25.23 ± 3.78^f^

Data represent the Mean ± standard error of three independent determinates. Mean within a row that did not differ significantly at 5% level of significance when compared with Fisher's least significant difference are followed by the same superscript letters.

### 3.3 Edible bamboos and their distribution in India

The choice of species in bamboo is primarily dependent on the needs of farmers, especially those in rural communities. Many species of bamboo are edible (Table 2), and the tribal communities mainly collect the bamboo shoots from natural forest areas. Many farmers also raise bamboo on their farmlands as a source of bamboo shoots.

**Table 2:** Different species of edible bamboos of India.

**Species**	**Region-wise distribution**	**References**
*Bambusa arundinacea* (Retz.) Willd.	North eastern region, Eastern region	(34, 54, 56)
*B. balcooa* Roxb.	Throughout India	(34, 54, 57, 58)
*B. bambos* (L.) Voss	Throughout India	(34, 54, 57, 58)
*B. khasiana* Munro	North eastern region	(54, 56)
*B. longispiculata* Gamble ex Brandis	North eastern region	(54)
*B. multiplex* (Lour.) Raeusch. ex Schult. and Schult. f.	North eastern region	(54)
*B. nutans* Wall. Ex Munru	North eastern region, Eastern region	(35, 54)
*B. pallida* Munru	North eastern region	(54)
*B. polymorpha* Munru	North eastern region, Eastern region, Central India	(35, 54)
*B. teres* Buch.- Ham. Ex Munru	North eastern region	(54)
*B. tulda* Roxb	North eastern region	(54, 59)
*B. vulgaris* Schrad. Ex Wendi	Throughout India	(54, 57, 58)
*Chimonobambusa Hookerianus* (Munru) Nakal. Synonym *Himalayacalamus Hookerianus* (Munru) Stapleton	North eastern region	(54, 56)
*Dendrocalamus asper* (Schult. and Schult. f.) Backer ex K. Heyne	Throughout India	(34, 35, 54, 57)
*D. brandisii* (Munru) Kurz	North eastern region	(54, 56)
*D. giganteus* Munru	North eastern region	(54, 56, 60)
*D. hamiltonii* Nees et Am. Ex Munru	North eastern region, southern India	(54, 56, 61)
*D. hookeri* Munru	North eastern region, southern India	(54, 56)
*D. longisphathus* Kurz	North eastern region	(54)
*D. sikkimensis* Gamble	North eastern region	(54, 56, 59)
*D. strictus* (Roxb.) Nees	Throughout India	(34, 35, 54, 57, 58)
*Gigantochloa albociliata* (Munru) Kurz	North eastern region	(54, 56)
*G. apus* (Bl. Ex Schult. F.) Kurz	North eastern region, other parts of India	(54, 56, 71)
*G. atroviolacea* Widjaja	Eastern region (West Bengal, Jharkhand)	(8, 35)
*G. macrostachya* Kurz	North eastern region	(54, 56)
*Himalayacalamus falconeri* (Hook. F. ex. Munru) Keng	North eastern region, Northern India	(54, 56)
*Melocanna baccifera* (Roxb.) Kurz	Throughout India	(35, 54, 57, 58)
*Phyllostachys bambusoides* Sieb. and Zucc.	North eastern region	(54, 56)

### 3.4 Nutritional composition of bamboo shoots and its safety

Bamboo shoots are highly nutritious and rich in both macronutrients and micronutrients. The moisture content in raw bamboo shoots varies significantly among species, ranging from 54.00% in *Bambusa arundinacea* to 94.70% in *B. nutans* (40). When compared among most of the edible bamboo species, bamboo shoots were found to contain substantial amounts of essential nutrients (Table 3). Among the macronutrients, the protein content in bamboo shoots ranges from 1.80% to 25.84% (40, 41), with the highest protein content of 25.84% recorded in *Bambusa balcooa* (42). The amino acid content ranges from 3.11% in *Dendrocalamus asper* to 3.98% in *B. bambos* (43). Bamboo shoots also contain oxalic acid in the range of 157 to 462 mg per 100 g of shoots (40), while the acidity content in different species ranges from 3.30% to 5.20% (23).

**Table 3:** Nutrient contents in 100 g fresh bamboo shoots.

**Nutrients**	**Range**	**References**
**Macronutrients**		
Protein (%)	1.80–25.84	(40–42)
Amino acid (%)	3.11–3.98	(43)
Oxalic acid (mg/100 g)	157–462	(40)
Acidic content (%)	3.30–5.20	(23)
Carbohydrate (%)	2.00–9.94	(40, 44)
Calories (Kcal/100 g)	14.00 – 27.00	(40)
Crude fiber (%)	23.10–35.50	(23, 40)
Fat (%)	0.30–3.97	(40)
Ash (%)	0.80–3.70	(23, 41)
**Micronutrients**		
**A. Vitamins**		
Vitamin A (I.U.)	20.00	(62)
Thiamine (Vitamin B1) (%)	0.05	(63)
Riboflavin (Vitamin B2) (%)	0.01	(63)
Niacin (Vitamin B3) (mg/100 g)	0.20–14.92	(64, 65)
Pyridoxine (Vitamin B6) (mg/100 g)	0.53–1.70	(65)
Vitamin C (%)	3.00–23.00	(23, 44)
Vitamin E (%)	0.61–0.91	(20)
α-tocopherol (mg/100 g)	0.26	(66)
γ -tocopherol (mg/100 g)	0.42	(66)
β-carotene (μg/100 g)	1.90	(66)
Lutein (μg/100 g)	35.60	(66)
**B. Minerals**		
Calcium (mg/100 g)	0.36–1,900	(23, 40)
Magnesium (mg/100 g)	5.38–140	(23, 44, 48)
Phosphorous (mg/100 g)	40–1,000	(23, 44, 62, 64, 67)
Potassium (mg/100 g)	20–1,400	(23, 43, 44, 65)
Sodium (mg/100 g)	8.22–400.00	(44, 48)
Iron (mg/100 g)	0.10–3.37	(43, 62, 67)
Selenium (mg/100 g)	0.0003	(48)
Zinc (mg/100 g)	0.57–1.01	(43)
Antioxidant activity (%)	13.97–46.00	(42, 68)

The carbohydrate content in fresh bamboo shoots ranges from 2.00% in *Melocanna baccifera* to 10.00% in *Bambusa arundinacea* (40, 44). The calorific value of bamboo shoots varies from 14.00 to 27.00 Kcal per 100 g of shoots (40). Previous studies have also revealed that bamboo shoots are a good source of fiber. For instance, *Phyllostachys bambusoides, D. hamiltonii, D. giganteus*, and *M. baccifera* contain crude fiber of 23.10%, 25.40%, 27.60%, and 35.50%, respectively (23). Additionally, bamboo shoots are known for their very low fat content, ranging from 0.30% to 3.97% in fresh shoots, with the maximum fat content recorded in *B. tulda* (40). The ash content in bamboo shoots also shows variation across species, ranging from 0.80% in *B. vulgaris* (41) to 3.70% in *Teinostachyum wightii* (23).

Fresh bamboo shoots contain Calcium ranging from 0.36 to 1,900 mg/100 g, Magnesium from 5.38 to 140 mg/100 g, Phosphorus from 40 to 1,000 mg/100 g, Potassium from 20 to 1,400 mg/100 g, Sodium from 8.22 to 400.00 mg/100 g, Iron from 0.10 to 3.37 mg/100 g, Selenium at 0.0003 mg/100 g, Zinc ranging from 0.57 to 1.01 mg/100 g, and Antioxidant activity ranging from 13.97% to 46.00%. However, bamboo shoots also contain cyanide (HCN), with the highest concentration recorded at the tip portion of the shoot (120 mg/kg), and followed by the middle portion (12 mg/kg) and the base portion (1.1 mg/kg) (45). Despite this, these concentrations remain within the permissible safe limit for human consumption, which is 10 mg HCN equivalent per kg dry weight, as recommended by FAO (46). Given this, the basal portion of bamboo shoots, which is typically soft and tender, is considered more preferable for consumption. Furthermore, proper processing techniques, such as slicing the shoots and boiling them for at least 15 min, can significantly reduce the cyanogenic content, with a maximum reduction of up to 91% (47).

### 3.5 Effect of harvesting age and fermentation on nutritive qualities of bamboo shoots

The harvesting age of bamboo shoots has a significant influence on both their nutritional and anti-nutritional components. While edible bamboo shoots are considered nutritionally rich, they also pose several challenges related to their safe consumption. One of the primary concerns is the presence of cyanogenic glycosides, especially taxiphyllin, which releases hydrogen cyanide (HCN) upon enzymatic hydrolysis. Without proper processing, either typically through boiling or fermentation, these compounds can cause acute toxicity (48). Additionally, bamboo shoots are considered goitrogenic due to their potential to interfere with iodine absorption, thereby affecting thyroid function (49). Their highly perishable nature and seasonal availability further limit their accessibility and utilization in many regions (50).

To address these issues, a combination of traditional and scientific processing methods is essential to ensure food safety and nutritional quality. In an experiment conducted by Pandey and Ojha (51), a considerable decline in protein and total phenol content was observed in shoots of *Dendrocalamus asper, D. strictus*, and *Bambusa tulda* when harvested at different growth stages. However, dietary fiber and carbohydrate content increased with the age of the shoots. Based on their findings, they recommended optimum harvesting ages of 10–14 days, 6–10 days, and 10–16 days (after emergence) for *D. asper, D. strictus*, and *B. tulda*, respectively. In many rural areas, tribal communities prefer consuming bamboo shoots in fermented form. Studies by Bam and Malagi (52) revealed that fermentation has a direct impact on the nutritive qualities of bamboo shoots. While fat and ash content decrease due to fermentation, sun-dried fermented bamboo shoots show an increase in crude fiber and protein content by 10.76% and 15.20%, respectively.

### 3.6 Socio-economic significance of bamboo shoots and market linkages

There is a significant demand for both fresh bamboo culms and processed bamboo shoots among local communities. Globally, the demand for bamboo shoots is also increasing rapidly. According to the Trade Overview 2014 by INBAR, India exported bamboo shoots worth 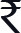 8.95 million but imported bamboo shoots worth 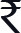
131.13 million (53). Although bamboo shoots are consumed in many Indian states, such as Odisha, Chhattisgarh, Jharkhand, Uttar Pradesh, and Kerala, the majority of production and consumption is reported from the northeastern states. Bhatt et al. (54) reported that edible bamboo shoots are predominantly harvested from the 1^st^ week of June to the 1^st^ week of September each year for consumption. In Northeast India, the average annual consumption of bamboo shoots in Arunachal Pradesh, Manipur, Meghalaya, Mizoram, Nagaland, and Tripura is reported to be 1,997, 2,188, 442, 433, 442, and 201 tons, respectively (54). In certain states like Meghalaya, Mizoram, and Sikkim, the tender edible shoots of *Dendrocalamus hamiltonii* are consumed the most, with an annual consumption of 483.81 tons, followed by *Melocanna baccifera* (361.06 tons), *Bambusa balcooa* (52.56 tons), and *Chimonobambusa hookeriana* (3.70 tons) (54).

In the existing marketing chain, traders and wholesalers play a vital role in the sale of bamboo shoots. Sakhrie and Sharma (55) observed that the highest marketing efficiency and producer's share in the consumer price were recorded when there was a direct producer-consumer business channel. However, many farmers and tribal communities engaged in bamboo shoot production lack adequate knowledge of modern technologies for plantation management, harvesting, processing, and marketing. Selling tender bamboo shoots provides a substantial source of income for tribal communities. For instance, in Meghalaya, Mizoram, and Sikkim, tribal communities were earning net revenues of 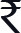
11.38 lakh, 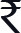 7.74 lakh, and 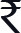 7.01 lakh per year, respectively, from bamboo shoot sales (54).

Nevertheless, inadequate transportation infrastructure often limits their ability to market their products effectively. Therefore, there is a need to establish a robust market network or bridge between producers and the supply chain. In many regions, the role of local markets and farmers' linkages to diverse bamboo products remains unexplored, with no location-specific product portfolios available. Additionally, the availability and accessibility of various promotional programs should be enhanced, particularly through the involvement of local agencies or NGOs, to meet the growing demand for bamboo shoots.

### 3.7 Socio-economic significance and market linkages of bamboo shoots in Tripura

#### 3.7.1 Income generation and livelihoods

Bamboo cultivation (including shoots) is increasingly seen as a profitable alternative to crops like rubber in parts of Tripura. Farmers report that income from bamboo (mature culms and shoots) supports their families and their children's education.

#### 3.7.2 Nutritional and cultural importance

Bamboo shoots are a staple in tribal diets and are valued for flavor, nutrition, and tradition. Both tribal and non-tribal populations widely consume them. Local cuisines include dishes such as *chakhwi awanduru, hontalia, and mosodeng*, which often feature bamboo shoots as a key ingredient.

#### 3.7.3 Key bottlenecks and opportunities

• ***Bottlenecks***

i. Legal/regulatory restrictions on harvesting from forests.
ii. Lack of consistent cold-chains or storage for shoots, leading to loss or reduced quality (Implied in need for processing/packaging infrastructure). Market access and unreliable demand for fresh shoots; competition with imported or non-indigenous varieties.

***Opportunities***

i. Expanding commercial cultivation of bamboo shoots (not only forest extraction), especially by tribal farmers.
ii. Better value addition (packaging, cleaning, branding) to fetch higher margins (The Times of India ± 1).
iii. Institutional support (TBM, state policy) to link growers to markets, strengthen clusters, apply modern technologies, and improve species/varieties.

### 3.8 Research gaps and route map for edible bamboo shoots

Despite the growing recognition of edible bamboo shoots as a sustainable nexus of nutrition, livelihood, and food security, several research and development gaps remain unaddressed. Comprehensive studies on varietal suitability, nutrient variability among species, and regional adaptation to diverse agro-ecological conditions are still limited. There is also a lack of standardized protocols for detoxification, post-harvest handling, and value addition, which constrains commercial-scale utilization. Furthermore, the absence of robust market infrastructure, farmer cooperatives, and policy integration restricts the economic potential of bamboo-based enterprises. Future research should prioritize biotechnological interventions to mitigate cyanogenic toxicity, develop cost-effective preservation and processing techniques, and improve the nutritional quality of bamboo shoots through breeding and agronomic optimization. Establishing community-based bamboo agroforestry models and integrating them into rural development and climate adaptation programs can serve as an effective route map. Strengthening linkages between research institutions, policymakers, and local stakeholders will be crucial to develop a holistic value chain—from sustainable cultivation and safe processing to market expansion and livelihood improvement—thereby positioning edible bamboo shoots as a cornerstone of India's bio-economy and sustainable food systems.

### 3.9 Conclusion and future perspectives

Edible bamboo shoots hold immense promise as a sustainable resource linking nutrition, livelihood, and ecological resilience in rural India. Their rich nutritional profile and compatibility with traditional food systems make them a valuable supplement for addressing malnutrition and enhancing food security. Integrating bamboo into agroforestry systems not only improves soil fertility and biodiversity but also contributes to carbon sequestration and rural income generation. However, challenges such as cyanogenic toxicity, short shelf life, and inadequate value-chain infrastructure hinder their wider adoption. Future strategies should focus on developing efficient post-harvest processing, storage, and value addition technologies, along with strengthening market networks and cooperative models. Policy support, research on safe processing methods, and community-level capacity building can together unlock the full potential of edible bamboo shoots, positioning them as a vital component of sustainable agriculture, rural development, and climate-resilient food systems in India.
